# COVID-19-related acute necrotizing encephalopathy with new spectroscopy features

**DOI:** 10.1590/0037-8682-0275-2022

**Published:** 2022-09-30

**Authors:** Guilherme Soares de Oliveira Wertheimer, Marcelo Barciela Brandão, Fabiano Reis

**Affiliations:** 1 Universidade Estadual de Campinas, Departamento de Radiologia e Diagnóstico por Imagem, Campinas, SP, Brasil.; 2 Universidade Estadual de Campinas, Departamento de Pediatria, Unidade de Tratamento Intensivo Pediátrica, Campinas, SP, Brasil.

A male 2-month-old infant presented with irritability, nasal discharge, and fever. During hospitalization, he had epileptic seizures and reduced consciousness and was transferred to an intensive care unit. Nasopharyngeal secretion polymerase chain reaction (PCR) was positive for SARS-CoV-2, and cerebrospinal fluid (CSF) analysis revealed elevated protein levels (78 mg/dL) and a negative viral panel. Interleukin analyses of serum and CSF were not available. An electroencephalogram showed multifocal paroxysmal activity. 

**FIGURE 1:**
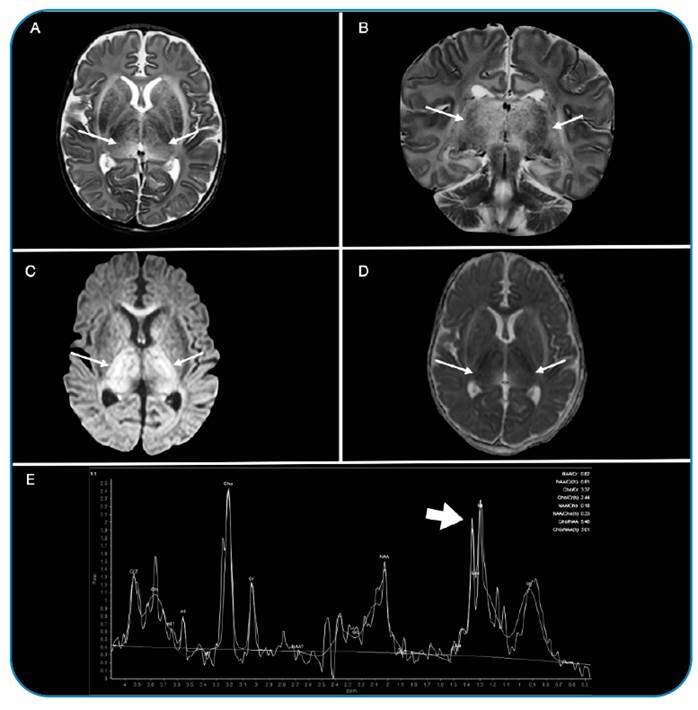
A two-month-old male tested positive for COVID-19. MRI shows restricted diffusion and bilateral tumefactive lesions of the thalamus and basal ganglia (arrows). MRI spectroscopy of the left thalamic lesion reveals lipid and lactate peaks (arrow) indicative of necrosis and anaerobiosis. This pattern is consistent with acute necrotizing encephalopathy. **ANE:** acute necrotizing encephalopathy; **MRI:** magnetic resonance imaging.

Brain magnetic resonance imaging (MRI) showed bilateral tumefactive hemorrhagic lesions of the thalamus as well as basal ganglia lesions, and spectroscopy revealed lipid and lactate peaks, a pattern consistent with acute necrotizing encephalopathy (ANE) (Figure 1). The patient showed marked clinical improvement after treatment with steroids and immunoglobulins. 

ANE is a rare encephalopathy mostly described in children and related to infectious agents, such as influenza-A, herpesvirus-6, and mycoplasma^1^. Its etiology remains unclear, but it is suspected to be an immune-mediated process involving *pro-inflammatory cytokines* (cytokine storm)^1^ ^,^ ^2^. ANE has a distinct neuroimaging pattern comprising changes in the thalamus, medial temporal lobe, pons, medulla, and, to a lesser extent, the striatum and subcortical perirolandic regions. ANE also involves variable hemorrhagic components and is sometimes found with coronavirus disease 2019 (COVID-19) infection^2^. In the present case, we detected a lactate peak on MRI spectroscopy, which has been described previously in ANE^3^ but not in cases related with COVID-19.
